# SSEP retains its value as predictor of poor outcome following cardiac arrest in the era of therapeutic hypothermia

**DOI:** 10.1186/s13054-019-2576-5

**Published:** 2019-10-23

**Authors:** Ted L. Rothstein

**Affiliations:** Department of Neurology, George Washington University, Washington, DC USA

**Keywords:** Critical care, Cardiac arrest, Cardiopulmonary resuscitation, Prognosis, Somatosensory evoked potentials, Therapeutic hypothermia

## Abstract

**Objectives:**

To re-evaluate the role of median nerve somatosensory evoked potentials (SSEPs) and bilateral loss of the N20 cortical wave as a predictor of unfavorable outcome in comatose patients following cardiac arrest (CA) in the therapeutic hypothermia (TH) era.

**Methods:**

Review the results and conclusions drawn from isolated case reports and small series of comatose patients following CA in which the bilateral absence of N20 response has been associated with recovery, and evaluate the proposal that SSEP can no longer be considered a reliable and accurate predictor of unfavorable neurologic outcome.

**Results:**

There are many methodological limitations in those patients reported in the literature with severe post anoxic encephalopathy who recover despite having lost their N20 cortical potential. These limitations include lack of sufficient clinical and neurologic data, severe core body hypothermia, specifics of electrophysiologic testing, technical issues such as background noise artifacts, flawed interpretations sometimes related to interobserver inconsistency, and the extreme variability in interpretation and quality of SSEP analysis among different clinicians and hospitals.

**Conclusions:**

The absence of the SSEP N20 cortical wave remains one of the most reliable early prognostic tools for identifying unfavorable neurologic outcome in the evaluation of patients with severe anoxic-ischemic encephalopathy whether or not they have been treated with TH. When confounding factors are eliminated the false positive rate (FPR) approaches zero.

## Background

Prognostication is a constant challenge for medical science. Predictive algorithms depend on historical information, but are only as reliable as the data that informs them. Having an accurate and timely technique which allows for early outcome prediction in those patients who remain comatose after CA is critical for allocating critical resources to those who would benefit, and provides realistic expectations and closure to those families whose loved ones have no hope of recovery.

Somatosensory evoked potential (SSEP) is just such a tool [1–14]. It is an objective, non-invasive, and inexpensive bedside technique that can be more sensitive than a detailed neurological examination [15]. SSEP is useful in assessing synaptic transmission within the central nervous system and includes cortical integrity [16]. SSEPs are elicited by electrical stimulation of the median nerves at the wrists and are thought to be the result of summated action and synaptic potentials from successive anatomic neural generators within the dorsal columns and thalamo-cortical sensory system [15]. Prior to the TH era, SSEP was heralded as the most reliable laboratory test for predicting unfavorable neurologic outcome following CA [1–3].

SSEP was identified *as* the key practice parameter of the American Academy of Neurology (AAN) published in 2006 [17].

However, recent publications have raised doubts of the reliability of SSEP as a predictor of poor outcome in the era of TH following cardiac arrest [18, 19]. Amorim and colleagues have compiled a number of isolated case reports which purport to show that the bilateral loss of the N20 cortical response can no longer be considered an infallible predictor of neurologic outcome [18]. Additionally, Howell et al. reported that in a retrospective study of 113 patients admitted to an inpatient rehabilitation center in anoxic-ischemic coma, 30% had “malignant SSEP results” [19]. The false positive rate to predict unfavorable outcome among those treated with TH was reported to be as high as 29%.

The patients cited in the case reports of both these small series have significant methodological limitations. They are lacking in essential information and, as will be shown, are seriously flawed. Moreover, these reviews do not comply with fundamental recommendations for data reporting as outlined in PRISMA [20].

There are many confounders in these published reports of patients with severe post anoxic encephalopathy who recover despite having lost their cortical N20 potential, which undermine their conclusions. These include insufficient clinical or neurological patient data, lack of detailed specifics of electrophysiologic testing, technical issues such as background noise artifacts, flawed interpretations (sometimes related to interobserver inconsistency), and the difference in interpretation and quality of SSEP among various clinicians and hospitals. Patients whose circumstances at the onset of CA were unknown may have had accidental “deep” core body hypothermia which went unrecognized.

**Fig. 1:**
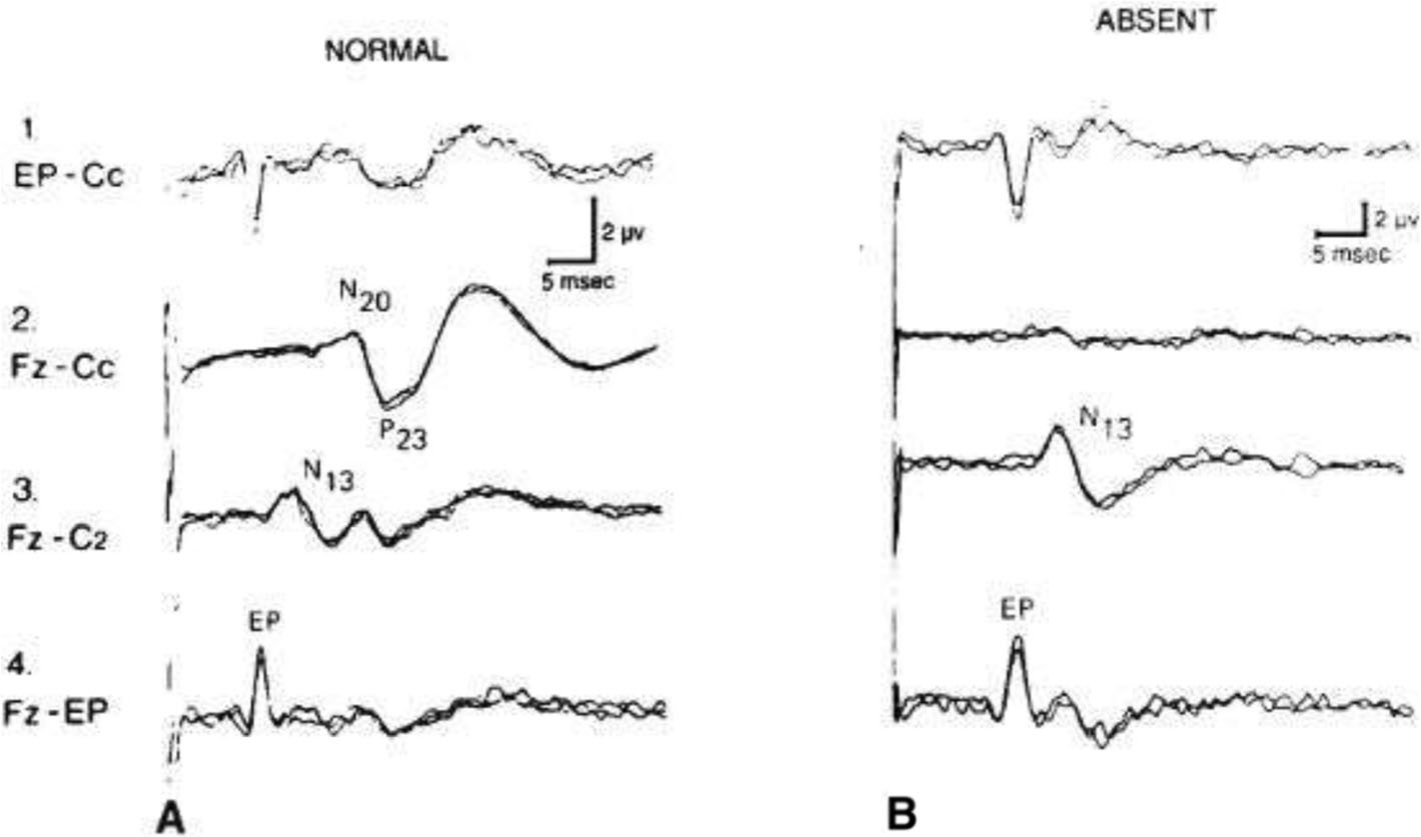
Somatosensory evoked potentials recorded from the scalp and neck of a normal subject after median stimulation at the wrist (**a**) and from a 78-year-old male with absent cortical responses after cardiac arrest who died without awakening (**b**). In **b** there is preservation of the brachial plexus (EP) and cervical medullary activity (N13) but N20 is absent in the Fz referenced contralateral cortex (Fz-Cc) as recorded in channel 2. In addition to pseudolaminar necrosis of the cortex, there was severe neuronal loss in the thalamus at necropsy

Rothstein and colleagues presented autopsy findings in 10 patients who died following CA [1] (Fig. 1). Each of 7 patients with bilateral absence of cortical evoked response had generalized necrosis of the cerebral cortex leading to the conclusion that there were no viable neurons capable of responding to an afferent stimulus [1, 6].

In another study, post mortem histologic analysis from 11 non-surviving patients following CA disclosed changes from hypoxic brain injury to the cortex with extensive cellular degeneration and shrinkage of neuronal nuclei and some with involvement of deeper subcortical structures including the basal ganglia. Each patient with moderate to severe thalamic damage had lost their cortical SSEP [21]. In another study, the earliest changes associated with anoxic-hypoxic brain damage were ischemic neuronal necrosis identified in cortical layers 3, 5, and 6 after only 5 h following CA [22].

Human bodies function within a narrow, carefully maintained core body temperature range [23]. Hypothermia is defined as core body temperature lower than 35 °C. When hypothermia progresses, organ systems can no longer work properly. As body cooling takes place, metabolic rates fall and neural transmission is inhibited. Other derangements include vasoconstriction, increased blood viscosity, and reduced tissue oxygenation [23]. Among 14 adult patients undergoing cardiac surgery, SSEPs were recorded at 1–2 °C steps as body temperature was lowered from 37 to 20 °C to determine temperature-dependent changes [24]. The cortically generated N20 wave disappeared between 20 and 25 °C. The N10 and N14 waves were selectively preserved being less vulnerable and therefore resistant to effects of hypothermia*.* Accidental hypothermia (AH) is a cause of cardiac arrest, and the risk greatly increases if core temperature is less than 28 °C (deep hypothermia) [25, 26]. AH can be caused by environmental exposures and various diseases which alter the thermoregulatory responses including trauma, sepsis, intoxications, and among the homeless [25–27]. AH can occur during any season and in any climate [25, 26]. Deep hypothermia with circulatory arrest was identified in 46 of 234 patients with accidental hypothermia [28]. Among those cases cited by Amorim and Howell whose clinical information regarding circumstances at the outset of CA are sparse, AH is possible, and temperature related changes must be taken into consideration [18, 19]. Standard thermometers are inadequate to this task, and as a result, temperatures below 35 °C are usually overlooked and missed [23]. Esophageal temperature probe is the most reliable and minimally invasive technique for measuring core temperatures [29, 30].

There is extreme variability in the quality and interpretation of SSEP recordings among clinicians and different hospitals. Pfeifer et al. conducted a retrospective investigation of the interobserver variability effect on the prognostic value of SSEP in CA survivors [31]. This study arranged for recordings of SSEP to be independently evaluated by 4 expert neurologists with “great experience” in the field. The mean correct prediction of SSEP for patients with an unfavorable neurologic outcome was 63%. One example was that of a patient diagnosed with absent N20 cortical responses whose revised evaluation identified a misinterpretation caused by artifact. Another retrospective study of SSEP was conducted upon 3 patients with good outcome who had SSEP initially classified as bilaterally absent. The analysis from 2 blinded neurophysiologists disclosed that noise in the registration made the results uninterpretable [32].

A further assessment of interobserver variability was undertaken by Zandbergen and colleagues [33]. SSEPs were interpreted independently by 5 neurophysiologists who were kept uninformed about the clinical status of 56 patients, other than their having anoxic-ischemic coma. Agreement among all 5 was only “moderate” as to whether the N20 cortical potential was present or not. The main source of disagreement related to the noise level, presence or absence of the cervical N13 wave, and low voltage negative waves. Sandroni et al. emphasize that in the ICU environment, evoked potentials are prone to electrical interference, which is the most important cause of interobserver variation [13].

## Discussion

In 2002, two randomized controlled trials on the use of mild TH demonstrated increased survival and improved neurologic outcomes in patients with out-of-hospital cardiac arrest [34, 35]. Induced hypothermia is associated with reduced levels of proinflammatory cytokines and free radicals, decreased permeability of the blood brain barrier, reduced neuronal excitation, and anticoagulant effects [36–40]. Their standard protocol calls for cooling patients to a target temperature of 32–34 °C where they are maintained for at least 24 h using surface or endovascular cooling techniques. However, a recent study of 950 patients who sustained out-of-hospital CA found that a targeted temperature of 33 °C did not confer any additional benefit compared with patients receiving a temperature of 36 °C [41].

The introduction of induced targeted therapeutic hypothermia (TTH) has become widespread as the standard of care for CA [42–46]. This has led to questioning the accuracy of traditional prognostic tools such as SSEP in TTH treated patients [18, 19, 47, 48].

Leithner and colleagues reported one case of recovery among 36 comatose anoxic-ischemic patients treated with TH who had bilaterally absent N20 components on the third day post arrest [48]. The patient was an alcoholic male found down and resuscitated within 10 min, who eventually fully recovered. A repeat SSEP performed 18 months later was normal. The case was disputed as there was only sparse clinical information on this sole survivor [49]. In responding to a letter questioning whether technical factors or brain trauma could have contributed to the result, Leithner et al. provided no new clinical details but denied that technical factors could have influenced the result [50]. One can assume that no imaging findings were obtained to exclude head trauma. It has been shown that malignant SSEP has a more favorable prognosis in patients with brain trauma compared to anoxic-ischemic coma, as those with absent N20 were found to have 10.2% chance of regaining awareness when brain swelling and hemorrhage resolve [51]. However, Blondin and Greer declared that due to Leithner’s single case of recovery, “a bilaterally absent N20 response at 72 h may not predict poor prognosis with absolute certainty” [47].

There are more recent additional reports of patients awakening despite malignant SSEP—some with minimal or no deficits, throwing doubt on the reliability of the absent N20 wave on SSEP as an infallible prognosticator of unfavorable outcome [18, 19]. Amorim et al. performed a meta-analysis of 35 articles on CA prognostication and uncritically identified 14 among 594 patients with an absent SSEP who recovered with relatively good functional outcomes [18]. They concluded that there is a false positive rate (FPR) for absence of the N20 in predicting poor neurologic outcome at 7.7% (95% Cl, 4–13%). However, methodological limitations can be raised with each of the patients cited in their analysis, which tend to contest Amorim’s validity in rejecting the SSEP as a predictor of negative outcome. The 14 cases that are identified in their paper will be addressed in detail.

Included in their text are the 3 cases of Bouwes et al. which were subsequently refuted by the authors, as well as the patient of Leithner et al. discussed above [32, 48].

The following cases are drawn from the remainder of the article’s depiction of subject characteristics for CA survivors with good outcome and bilaterally absent SSEP [18] (Table 1).

**Table 1:** Summary of clinical characteristics (when known) and details of recovery in survivors of cardiac arrest reported to lack N20 responses on somatosensory evoked potentials. In each case, the false positive result is disputed

References	Outcome (timing after ROSC)	Temperature management	Onset known	SSEP	Clinical data	Authors’ conclusions or limitations
Arch et al. [52]	Ambulate with walker, dysarthric speech at 22 days	TH to 32–34 °C	Unknown	Absent N20 at 49 h	31-year-old CA “victim” Sluggish pupils No response to pain	Inadequate clinical data AH possible at the outset No core body temp
Bender et al. [53]	CPC 2 at 6 months	No TH	+CA while playing soccer	Non-standard montages at days 3 and 9	16 years old No data	No clinical data SSEP not reliable
Bouwes et al. [32]	3 cases	TH to 32–34 °C	No data	“Technical difficulties”	No data	FPR “refuted” by authors
Codeluppi et al. [54]	Normal neurologic examination	TH at 33 °C × 24 h	+Multi-drug overdose	Uninterpretable: “noise” at 84 h	34-year-old M with GCS 4	AH possible No core body temp SSEP: “noise”
Dragancea et al. [55]	CPC 2 at ICU discharge, CPC 1 at 6 months	TH at 36 °C	No data	“Technical artifacts” at 77 h	No data	SSEP “interpretation difficult” No clinical data
Guerit et al. [56]	2 cases: GOS 4–5	No TH	+Anesthesia accidents	Misinterpreted as absent N20	One 25-year-old M made full recovery	SSEP in only figure shows attenuated, not absent N20
Howell et al. [19]	GOS 4–5 at 8 months	No TH	No data	Bilaterally absent N20 No SSEP figure to review	25-year-old M comatose for 1 week Recovery of Cs after 10 weeks	No clinical data AH possible No core body temp No SSEP to review
Karunasekara et al. [58]	CPC 2	No TH	+Attempted hanging	Figure 2 Uninterpretable: “noise”	51-year-old M GCS 3 Pupils sluggish At 6 days, response to pain	“Neck injury reduces validity of N20 SSEP use in prognostication” SSEP: “noise”
Leithner et al. [48]	Regained Cs with normal cognition at 18 months	TH at 36 °C	Alcoholic “found down”	Absent N20 at 3 days Normal SSEP at 18 months No SSEP montages	No clinical data	No head MRI/CT to evaluate for trauma No clinical data
Weinstein et al. [59]	Dysarthria Ambulates with cane at 6 months	TH at 33 °C × 24 h	No data	Absent N20 at 20 days Montages not recorded	36-year-old F No clinical data Opens eyes day 29 Follows commands day 31	No clinical data No SSEP montages for review
Young et al. [60]	GOS between 3 and 5 at 3 months	No TH	No data	No figure to review	Eventually recovered awareness	Author attributes to “Watershed ischemia”

*Abbreviations*: *AH* accidental hypothermia, *CA* cardiac arrest, *Cs* consciousness, *CPC* cerebral performance category score, *CT* computerized tomographic scan, *FPR* false positive response, *GCS* Glasgow Coma Scale, *GOS* Glasgow Outcome Score, *MRI* magnetic resonance imaging, *ROSC* return of spontaneous circulation, *SSEP* somatosensory evoked potentials, *TH* therapeutic hypothermia

Arch et al. describe a 31-year-old comatose male following CA with no information on the circumstances or etiology of his ventricular fibrillation [52]. Neurologic examination was limited to sluggishly reactive pupils, flaccidity, and lack of posturing to painful stimulation. No information is provided on the patient’s core body temperature.

Bender et al. identify a 16-year-old who sustained CA while playing soccer and was admitted in coma [53]. The patient had dilated pupils unresponsive to light. After 25 min of resuscitation, the pupils became reactive but he remained comatose. Heart rhythm showed ventricular fibrillation, with return of spontaneous circulation after 28 min. SSEP obtained on day 3 and repeated on day 9 disclosed no cortical response. However, standard SSEP montages were not utilized. Top 3 channel recordings are normally obtained from EP-Cc, Fz-Cc, and Fz-C2, rather than C7 in channel 2 [16]. Placement of electrodes can be a critical factor in obtaining accurate results.

Codeluppi et al. describe a 34-year-old drug addict who sustained CA after a cocaine and heroin overdose and received TTH [54]. SSEP performed after 84 h allegedly show bilateral absent cortical response. The Figure 1 in Codeluppi’s manuscript shows a series of waveforms at 84 h post arrest which are not diagnosable. There is no well-defined or consistent Erb’s point wave from either right or left side. The N9 wave identified is in the figure at 10 ms on the left and 7.5 ms on the right. Repeat SSEP at 13 days when cortical responses are present shows well defined Erb’s point on both sides at 12.5 ms. Deep hypothermia can occur with polysubstance abuse, but no core body temperature readings were performed in this instance [23, 27].

Dragancea et al. review the outcome in 313 patients with cardiac arrest treated with TTH who were prognostically assessed, and bilateral absence of the N20 peaks was found in 74 patients, among whom one patient had a good outcome [55]. However, the interpretation performed at 77 h post arrest was reported by the authors to be difficult due to “technical artifacts”.

Guerit et al. described two young patients in anoxic coma as a result of anesthetic accidents and studied on the day of their arrest with absent N20 potentials [56]. An additional patient with cerebral anoxia under similar circumstances was subsequently identified (personal communication, JM Guerit, October 18, 1999). Eventually, all 3 patients recovered consciousness and had a return of N20, but only one made full recovery (personal communication JM Guerit, February 24, 2000). A single subject had SSEP published recordings over a 3-day period. Review of the N20 potential on this patient was interpreted by authors as absent on the first day, but appears to be present although attenuated at P’_3_ and P’_4_ with increasing amplitude over the next few days.

Howell et al. performed a retrospective study of 113 patients admitted to a neurorehabilitation center in whom 22 recovered consciousness despite malignant SSEP [19]. There is clinical data on only one patient, a 25-year-old male not treated with TH, in a coma for the first week with a “malignant SSEP”. The patient began recovering consciousness 10 weeks after the initial CA. There are no SSEP recordings available for review, nor details of the electrophysiologic techniques used to perform the recordings. There is no data on the circumstances surrounding the occurrence of CA and core body temperatures were not obtained. Despite these limitations, Young, in an editorial response to the article, laments that *all* the predictors of poor outcome following cardiac arrest including SSEP have been shown to have a higher false positive rate than initially stated [57].

Karunasekara et al. describe a 51-year-old male who had a CA after a failed attempted hanging [58]. He underwent CPR for pulseless electrical activity rhythm. Immediate management consisted of intubation and assisted ventilation due to hypoxia. Despite the patient’s neck injury, a cervical MRI was not performed to define whether cervical cord injury had occurred (although brain MRI was performed). The alleged absent cortical potential as presented in Fig. 1 appears to be an example of noise. The SSEP in Fig. 1 reveals that neither Erb’s point, cervical N9, nor N13 are recorded. Importantly, there are no details of neurologic examination at the time SSEP was recorded. The authors acknowledge that with “distension and inflammation of the neck” there may have been disruption in the “nerve transmission pathway” and therefore not an indicator of severe cerebral injury.

Weinstein et al. identify a 36-year-old woman found to have pulseless ventricular tachycardia [59]. She was treated with TH to 33 °C for 24 h. No details are available as to the cause of her CA or her neurologic findings other than that she was unresponsive. An EEG revealed periodic epileptiform discharges. SSEP on post arrest day 20 disclosed an absent cortical response. No specifics are available as to the method of electrophysiologic testing. The channels used for the recording are not identified. The patient opened her eyes on day 29 and was able to follow commands on day 31.

Young et al. described a single patient among 20 in whom the N20 response was absent and recovered awareness [60]. No further details on the case are provided. Imaging studies were not performed, and the patient’s SSEP findings were subsequently attributed to “watershed ischemia” (Personal communication GB Young, January 6, 2012). In a more recent article authored by Young referring to a meta-analysis of 802 patients with bilateral absence of the N20 response, “there were no false positives” [61].

## Conclusions

Amorim’s claim that SSEPs are no longer infallible predictors of poor outcome, and that the FPR is several times higher than commonly accepted based on their review of 14 individual cases, does not withstand critical scrutiny [18]. Each of the patients identified in their report has flawed information on which their conclusions are based. None provides the necessary or sufficient information as examples of SSEP unreliability in predicting unfavorable neurologic outcome.

By way of contrast with these reports on the prognostic limitations of SSEP in CA patients, Sandroni et al. reviewed 50 studies with 2828 patients not treated with hypothermia who had absence of SSEP N20 wave at 24 h, which reliably predicted poor outcome early with no false positive responses [12]. A further study by Sandroni et al. reviewed 37 studies involving 2403 patients who received TTH with similar conclusions [13]. In both reports, during the first week following CA, a bilaterally absent N20 obtained with SSEP predicted unfavorable outcome with no false positive responses. Both studies reflect on the potential for bias as the absence of N20 cortical potential could lead to a decision to prematurely withdraw treatment resulting in a self-fulfilling prophecy.

Continued study of putative survivors of CA lacking in cortical evoked potentials is important. Such patients should have been thoroughly investigated with multiple modalities of prognostic assessment including detailed clinical and electrophysiological data, and have normal core body temperatures at the time of their SSEP recording. Such cases would justify the need, widely proposed and generally acknowledged, to exercise extreme caution using SSEP in isolation as sole determining factor for withdrawing life support to avoid self-fulfilling prophecy. This recommendation is in conformity with the European Resuscitation Guidelines which endorse the concept that the decision to limit care should not be based on the results of a single prognostic tool [45, 62]. However, the guidelines proposed prior to the TH era still hold, and SSEP can assist as a specific predictor of poor outcome. SSEP should be one of a number of investigations that clinicians can use when challenged on whether or when to withdraw life sustaining care from patients who remain comatose following CA [63–66].

In conclusion, the absence of the cortical N20 wave obtained with routine SSEP remains one of the most reliable and reproducible predictors of negative outcome, whose FPR approaches 0% [12–14].

## Data Availability

Not applicable to this paper.
